# Analysis of the Perception and Treatment of Osteoarthritis of the Knee Through Social Media: An Observational Study of the Top 100 Viral TikTok Videos

**DOI:** 10.7759/cureus.48487

**Published:** 2023-11-08

**Authors:** Thomas I Hong, Sophie L Bernstein, Alejandra Ramirez, Alex Gu, Amil R Agarwal, David M Lutton, Sean Tabaie

**Affiliations:** 1 Orthopaedic Surgery, George Washington University School of Medicine and Health Sciences, Washington DC, USA; 2 Orthopaedic Surgery, University of Missouri-Kansas City School of Medicine, Kansas City, USA; 3 Orthopaedic Surgery, Children's National Hospital, Washington DC, USA

**Keywords:** patient education, knee treatment, video content analysis, evidence-based practice, orthopaedic guidelines, medical misinformation, social media, tiktok, osteoarthritis

## Abstract

Background: TikTok is a popular platform that can be used for medical insights. However, spreading inaccurate information about diagnosing or treating medical conditions can undermine the quality of patient care. Our assessment focused on the discourse surrounding knee osteoarthritis on TikTok, with two primary objectives: 1) identifying the creators behind osteoarthritis-related content, and 2) examining whether a connection exists between the reach of video content and the strength of recommendations provided.

Methods: The top 100 TikTok videos were chosen based on likes on March 29, 2023. Posts were identified using the hashtag (“#Osteoarthritis”). Videos were classified by the following: number of likes, comments, shares, date of upload, uploader (medical professional, non-medical professional, or business), and video content (medical treatment, home remedy, personal story). Treatments were rated according to the American Academy of Orthopaedic Surgeons (AAOS) Evidence-Based Clinical Practice Guideline for Osteoarthritis using the strength of evidence criteria (1-4 stars). Descriptive and univariate analyses were performed.

Results: Among the top videos, 67.7% were uploaded by medical professionals. Private companies, despite having the highest average likes (29,681.2) and shares (1,367.5) per video, had a limited average evidence strength of 2.13. Physician-created videos had the second-highest average number of likes (25,440.1) and shares (1,224.5) per video with a strength of evidence of 3.03. Non-medical professional videos had the lowest evidence support (0.89). Medical treatments, the most liked and shared content, had the lowest evidence strength (1). There was no statistically significant correlation between the number of likes (p=0.808), comments (p=0.647), or shares (p=0.439) to the strength of evidence regarding the intervention.

Discussion: TikTok can be unreliable for knee osteoarthritis treatment information. It is common to find non-physicians sharing medical advice on the platform, with medical treatments demonstrating the weakest level of supporting evidence. Orthopaedic surgeons should advise their patients that TikTok treatment recommendations may not align with established guidelines.

## Introduction

Conventional patient-physician interactions no longer solely serve as patients' primary information source. While healthcare practitioners continue to be the foremost trusted experts for health-related knowledge, various social determinants influencing patients, such as geographical constraints, financial considerations, and time limitations, can lead to a preference for online research instead of in-person medical consultations [1]. Research has shown that as many as 80% of internet users seek health information online [1-3]. Social media platforms, driven by user-created content, have created a sense of virtual community, support systems, and a way to exchange information. Specifically, health and medical information is amongst the most popular themes researched when it comes to acne, weight, signs and symptoms of pain, and interventions [4,5].

The effectiveness and safety of these online treatment alternatives remain unverified. It is still uncertain whether these platforms have predominantly yielded positive or negative outcomes in a patient's treatment approach. While social media platforms have the capacity to contribute positively to a patient's well-being, incorrect usage can conversely lead to exacerbated health results, strained patient-physician interactions, and heightened healthcare disparities [4]. We recognize that user-generated content is susceptible to inaccuracies and biases, posing additional risks to individuals who lack the necessary medical literacy despite their digital competence. Irrespective of whether user-generated content holds health-related credibility or not, its presence on social media remains constant. The pervasive impact of social media is undeniable, significantly influencing millions of patients across the globe, thus rendering it an aspect that physicians cannot disregard.

This study focuses on one of the most popular and influential platforms, TikTok, which is a social media platform for sharing, generating, and consuming user-created content. Its model of vertical scrolling, short-form, video-based content is easy to comprehend, easily accessible, and searched utilizing hashtags to highlight key topics [6]. Since its U.S. launch in August 2018, the platform has experienced meteoric growth, already having 150 million monthly U.S.-based users as of February 2023 [7]. Given that video-based patient education has proven to be an established tool for improving patient satisfaction, physicians should explore using TikTok as a conduit for their patients to interpret medical information [8].

The potential virality and appeal of a video may significantly influence a patient's perception of the information presented on platforms like TikTok. Unfortunately, the widespread dissemination of inaccurate health information on social media has emerged as a serious public health concern. Health misinformation can be defined as health-related claims that rely on anecdotal evidence, are outright false, or misleading due to a lack of solid scientific backing [9]. This demonstrates the need for infodemiology, the science of managing health information, and effective communication about medical conditions in parallel with the expansion of social media.

To enhance the effective dissemination of medical education to the public, it becomes imperative to assess several key factors. This includes a thorough examination of health-related subjects shared on platforms like TikTok, encompassing information about medical treatments, diverse diagnoses, public awareness surrounding these diagnoses, and the credibility of the content creators themselves. This study specifically delved into the content present on TikTok that addresses the topic of knee osteoarthritis (OA). The primary objective was twofold: firstly, to scrutinize the makeup of content creators focusing on this subject, and secondly, to ascertain whether a correlation exists between the viewership of a video and the strength of the recommendations it provides.

## Materials and methods

**Figure 1:**
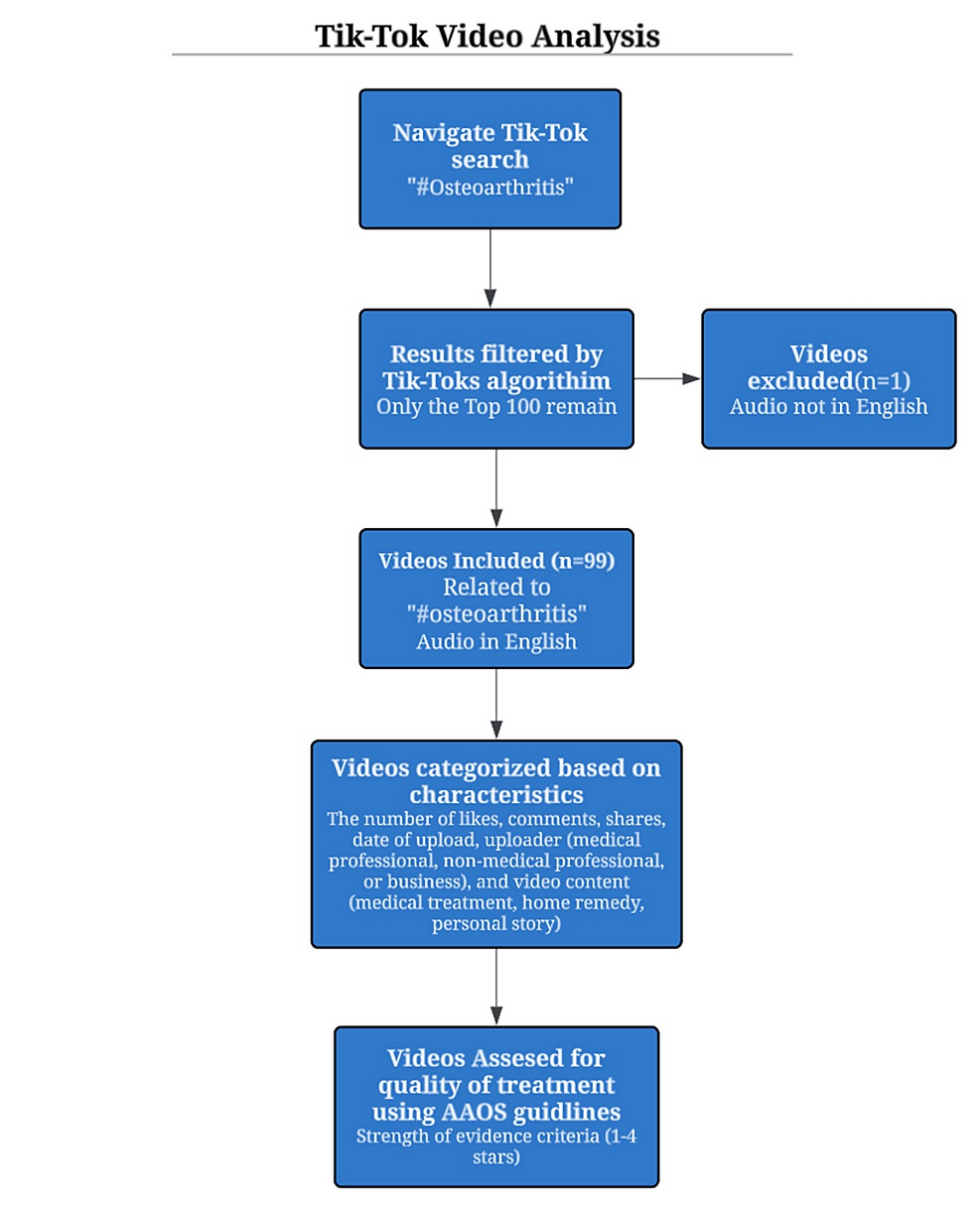
Observational Analysis Flowchart of TikTok Videos

This observational study involved the evaluation of TikTok posts on March 29, 2023. The data screening process, as illustrated in Figure 1, was guided by the American Academy of Orthopaedic Surgeons (AAOS) Evidence-Based Clinical Practice Guideline for Osteoarthritis. Beginning with the first step, the TikTok search engine was utilized to identify the top 100 publicly available videos associated with the hashtag ("#Osteoarthritis"). The assumption was made that these 100 videos, due to their viral nature, exerted the greatest influence on the audience. Among these top videos, 99 were in English, and one was in Spanish; the latter was excluded from our analysis. Next, the videos were categorized using Excel, considering factors such as the number of likes, comments, shares, upload date, uploader's profile (medical professional, non-medical professional, or business), and the video's content nature (medical treatment, home remedy, personal story). This breakdown aimed to provide insights into the type of uploaders wielding the most influence and the content they presented. Ultimately, the treatments suggested by users were classified based on their alignment with the approved treatment options defined in the AAOS Evidence-Based Clinical Practice Guideline for Osteoarthritis, applying a strength of evidence criteria ranging from 1 to 4 stars. Once all these categories were addressed, descriptive and univariate analyses were conducted to derive meaningful insights.

## Results

Of the top 100 videos, 99 were in English and analyzed. Of these videos, only 67 (67.7%) were uploaded by medical professionals. Table 1 provides a comprehensive breakdown of the number of videos by uploader type, including the average number of likes, comments, shares, and the strength of evidence. Private companies had the highest average number of likes (29,681.2) and shares (1,367.5) per video, but the strength of evidence was 2.13, which correlates to limited evidence supporting the intervention. Physicians’ videos had the second-highest average number of likes (25,440.1) and shares (1,224.5) per video and had a strength of evidence of 3.03 which correlates to moderate evidence supporting the intervention. The individual non-medical professional videos had the lowest strength of evidence for an uploader (0.89), correlating with little to no evidence-based intervention recommendations. Regarding content type, content described as medical treatments received the most likes and shares; however, it had a strength of evidence 1, which correlates to no evidence supporting the intervention. There was no statistically significant correlation between the number of likes (p=0.808), comments (p=0.647), or shares (p=0.439) to the strength of evidence regarding the intervention (Table 2).

**Table 1:** Characteristics of the Top 100 Most Popular TikTok Videos on Osteoarthritis

	Number of Videos	Average Number of Likes	Average Number of Comments	Average Number of Shares	Average Strength of Evidence
Uploader type					
Individual: Medical professional	67	12,333.50	145.2	644.6	3.33
Nurse	4	1,595.30	19.3	245.5	4
Occupational therapist	2	177.5	8	10	4
Physical therapist	29	1,063.00	30.9	145.3	3.57
Physician assistant	1	138	7	11	4
Physician	31	25,440.10	281.7	1,224.50	3.03
Individual: Non-medical professional	14	2,209.60	184.1	375.3	0.89
Private company	18	29,681.20	181.72	1,367.50	2.13
Content type					
Medical treatment	9	40,643.40	256.2	1,802.90	1
Home treatment	33	7,495.80	142.6	736	2.19
Personal story/awareness	57	13,655.90	150.2	570.9	3.73

**Table 2:** Strength of Evidence Relative to Study Variables

Strength of Evidence	p-value	R²
Number of likes	0.808	0.001
Number of comments	0.647	0.003
Number of shares	0.439	0.008

## Discussion

Social media has significantly influenced the landscape of healthcare communication, serving as a prominent source of medical information for patients. Both medical providers and patients extensively use these platforms, which serve as fast and accessible channels for sharing and seeking information about health-related topics [1,3,4]. Nearly 80% of internet users search for healthcare information online, and patients often turn to social media for information about their conditions, treatments, and healthcare providers [1-3]. Additionally, social media has been proven to be effective in enhancing knowledge, attitudes, and self-care activities, as demonstrated by previous programs for patients with diabetes [10]. These platforms also provide a forum for asking health-related questions and raising awareness about various diagnoses.

TikTok, the fastest-growing social media platform of the 2020s, sees increasing usage in medicine [6]. Yet, in the field of orthopaedic surgery, its utilization is surprisingly low. Chiang et al. analyzed 1,231 Pediatric Orthopaedic Society of North America (POSNA) members actively practising in the United States and found none of the members maintained an active TikTok account [11]. This finding highlights an untapped potential for this platform to enhance orthopaedic patient education and communication.

We probed this potential in our study by analyzing the TikTok content related to OA of the knee, a prevalent musculoskeletal condition. We examined content creators and explored the relationship between video reach and evidence strength. Our findings reveal a stark landscape of health information on TikTok, with non-physicians contributing most of the content. Of the searchable top 100 OA videos on the platform, physicians uploaded just 31 videos. In terms of video content, despite having the second lowest average strength of evidence (1.00), videos discussing medical treatments garnered the most likes (40,643.4) and shares (1,802.9). This far surpassed the personal stories or awareness videos that held the highest strength of evidence (3.73). These findings underscore the widespread issue of health misinformation, where misleading or false information often reaches a wider audience than fact-based, scientifically validated information. This is of particular concern because, on social media, false information routinely reaches between 1,000 and 100,000 people, whereas true information rarely extends beyond 1,000 people [9,12].

Our research suggests that TikTok, more often than not, proves to be an unreliable platform for obtaining information about knee osteoarthritis. This carries significant implications as it could potentially distort a patient's understanding of their disease. A previous study has shown that three-quarters of patients could accurately define osteoarthritis [13]. However, when faced with inaccurate or misleading information from sources such as TikTok, there is a risk that this understanding could be compromised. Furthermore, the progression of osteoarthritis may necessitate total knee arthroplasty, and studies have shown that patients’ preoperative expectations are pivotal in predicting improved functional outcomes and satisfaction following total joint arthroplasty [14]. In this light, the misinformation spread on social media platforms could adversely impact patient expectations and, therefore, their overall treatment outcomes.

In line with our findings, Anastasio et al. [15] also reported disappointing discoveries of orthopaedic information on TikTok. Their evaluation of 100 TikTok videos on ankle sprain exercises found only 2% scoring a "fair" grade, with none reaching "good" or "excellent," based on two different grading scales [15]. Similar poor findings were reported on the educational value of shoulder instability exercise videos on TikTok [16]. This evidence further emphasizes the need for the medical community to take action in addressing the misleading or inadequate health information circulating on social media platforms.

While our study highlights significant concerns around misinformation about OA on TikTok, the situation is not entirely bleak. We discovered that amongst videos created by medical professionals, physician-created content had the highest average number of likes (25,440.1), comments (281.7), and shares (1,224.5), suggesting that physician voices are amongst the most influential on the platform. This presents an opportunity for orthopaedic surgeons to participate on TikTok and redirect the narrative about musculoskeletal disease. Currently, the adoption of social media in orthopaedic subspecialties exhibits considerable variance, with only 37% utilization among shoulder and elbow surgeons, and approximately 64.7% among sports medicine specialists [17,18]. Clearly underutilized by physicians, TikTok could be a valuable platform for orthopaedic surgeons. It presents an opportunity not only to correct misconceptions but also to guide patients toward treatments that align with approved guidelines. If utilized effectively, TikTok has the potential to enhance patient engagement, enable remote follow-up visits, and promote surgeon education through real-time interaction, all without incurring additional costs [3,19].

However, our study has limitations. We focused on the most-liked videos, which may not necessarily represent the full range of content available on the platform. We are also aware that older generations suffering from OA may not have the TikTok application, narrowing our audience. Moreover, the TikTok algorithm, which potentially affects the visibility and popularity of videos, was not considered in our analysis.

Social media can be an innovative platform for disseminating and accessing health information if used with prudence. This powerful connectivity continues to allow users to post content that is public, rapidly accessible, and searchable by a global audience. Social media permeates our lives and, with its worldwide presence, may have profound consequences. The advent of TikTok and its integration into healthcare presents both opportunities and challenges for healthcare providers. While it offers a new method to educate patients about conditions such as OA, it also has the potential to spread misinformation. The key for patients to successfully utilize TikTok, and all social media platforms, lies in being able to understand the nature of the content and the reliability of its sources. We urge medical professionals to strongly consider the impact TikTok and other social media platforms have on patients. Given the close intimacy of social media and healthcare, it is important that physicians incorporate this relationship into their medical encounter and delivery of care with their patients. As our study finds, a physician's presence has the largest impact on social media and remains the most valuable point in the medical field. Consequently, with their tenable and trusted presence, both virtually and physically, physicians can profoundly decrease the threats to patients brought forth by misinformation on TikTok.

## Conclusions

TikTok, a rapidly growing social media platform, has become an influential tool in shaping health perceptions. This study showed that a significant proportion of information on the platform may not align with established orthopaedic guidelines for osteoarthritis of the knee. Videos by non-physicians, despite being influential, often lack robust evidence. However, content by physicians enjoys substantial viewership, indicating a pivotal role they can play in counteracting misinformation. Recognizing the influence and accessibility of TikTok, it is paramount for orthopaedic surgeons to actively participate in this digital arena, guiding patients towards evidence-based treatments and enhancing their understanding of conditions like osteoarthritis.
